# Efficacy of slow-coagulation cyclophotocoagulation after failed MicroPulse cyclophotocoagulation in refractory glaucoma

**DOI:** 10.1007/s10103-025-04631-4

**Published:** 2025-09-20

**Authors:** Baltaj S. Sandhur, Mohit Jethi, Natasha Gautam, Sinan Ersan, Logan Jay, Sandra F. Sieminski

**Affiliations:** 1 Department of Ophthalmology, Jacobs School of Medicine and Biomedical Sciences, Buffalo, United States; 2 Ross Eye Institute, Buffalo, United States; 3 Department of Ophthalmology, Jacobs School of Medicine and Biomedical Sciences, Buffalo, United States

**Keywords:** MicroPulse transscleral cyclophotocoagulation (MP-TSCPC), Slow-Coagulation continuous wave cyclophotocoagulation (SC-CWCPC), Refractory glaucoma, Iridex, Intraocular pressure (IOP)

## Abstract

Slow-coagulation continuous wave cyclophotocoagulation (SC-CWCPC) is a promising treatment option for refractory glaucoma. However, its efficacy following a failed MicroPulse transscleral cyclophotocoagulation (MP-TSCPC) procedure remains unexplored. This study evaluates the efficacy of SC-CWCPC in patients with uncontrolled intraocular pressure (IOP) after undergoing MP-TSCPC. A retrospective case series of patients with uncontrolled glaucoma who underwent SC-CWCPC following MP-TSCPC failure was conducted. All procedures were performed by a single glaucoma surgeon between January 2022 And October 2024. Patients with < 3 months follow-up were excluded. SC-CWCPC was performed using 1250-milliwatt power and 4-second applications. Surgical success was defined as IOP between 6 and 21 mmHg with a ≥ 20% reduction from baseline on topical medication, no incisional reoperation for glaucoma, and no loss of light perception vision. Eight patients (10 eyes) with a mean age of 43.75 ± 24.59 years were included. 90% (9/10) had prior Tube/Trabeculectomy procedures. Mean preoperative IOP was 26.10 ± 15.80 mmHg on 3.1 ± 1.20 antiglaucoma medications (AGM). At a mean follow up time of 10.97 ± 3.42 months, IOP decreased to 15.80 ± 5.31 mmHg (*p* = 0.002) with 2.30 ± 1.49 AGM (*p* = 0.070) with an overall success rate of 80%. One eye required Ahmed Glaucoma Valve implantation one year postoperatively while another had prolonged inflammation that resolved medically. No cases of persistent hypotony or significant visual acuity loss occurred. SC-CWCPC is an effective and safe surgical intervention in patients with refractory glaucoma following failed MP-TSCPC. The results of our study are helpful in expanding the role of the SC-CWCPC technique in the management of glaucomatous patients.

## Introduction

Cyclodestructive techniques aim to decrease intraocular pressure (IOP) through the destruction of the ciliary body, which is responsible for producing aqueous humor. The earliest forms of these procedures included cyclocryotherapy, cyclodiathermy, and cyclotomy of the ciliary body [1]. Concerns for the safety and efficacy of these procedures led to the development of transscleral cyclophotocoagulation (TSCPC). Laser energy applied directly to the sclera is absorbed by the pigmented epithelial cells of the ciliary body leading to coagulative necrosis, which is responsible for the decrease in IOP [2]. Since TSCPC’s introduction, various forms of cyclophotocoagulation have been developed, such as continuous wave cyclophotocoagulation (CWCPC) and the more recent MicroPulse transscleral cyclophotocoagulation (MP-TSCPC) [3]. 

Both CWCPC and MP-TSCPC utilize a laser to deliver energy to the ciliary body via a probe. Due to its on-off functionality, which provides short bursts of laser energy followed by a rest period, MP-TSCPC results in less thermal damage to the ciliary body and adjacent structures [4–6]. As a result, MP-TSCPC has a lower rate of vision threatening side effects such as phthisis, chronic inflammation, and hypotony compared to CWCPC. Despite its better safety profile compared to CWCPC, MP-TSCPC is still traditionally reserved for refractory glaucoma as referenced in the American Academy of Ophthalmology’s Primary Open-Angle Glaucoma Preferred Practice Pattern, with procedures such as trabeculectomy, tube shunts, and laser trabeculoplasty being the more common initial treatment [7]. More recent studies have demonstrated the efficacy and safety of MP-TSCPC in patients with good central vision, and as a result, some surgeons have utilized MP-TSCPC in patients they may have not considered CWCPC [8, 9]. 

In patients who have failed initial or repeat MP-TSCPC, the surgeon is not left with many options in refractory glaucoma, as they typically have exhausted all other options prior to attempting this. However, the surgeon may be reluctant to utilize the traditional high energy settings of CWCPC due to the side effect profile. That is where slow-coagulation continuous wave cyclophotocoagulation (SC-CWCPC) can be considered. SC-CWCPC is a technique that utilizes less energy for a longer duration per application, presumably causing less coagulative damage and less vision threatening side effects than traditional CWCPC. On its own, SC-CWCPC has been shown to be effective in treating refractory glaucoma [10, 11]. Prior studies evaluating SC-CWCPC in patients with refractory glaucoma found success ranging between 50.1% and 71.7% [10, 12]. 

A recent prospective study evaluating SC-SWCPC and MP-TSCPC in refractory glaucoma demonstrated a greater short-term reduction in mean IOP with slow coagulation [13]. While SC-CWCPC is an appropriate treatment for refractory glaucoma with promising results, its use specifically following a failed MP-TSCPC remains unexplored. Our study is the first to evaluate the long-term efficacy of SC-CWCPC in this setting. We hypothesize that SC-CWCPC following MP-TSCPC serves as an effective treatment option for those with refractory glaucoma who have failed MP-TSCPC, with a low side effect profile.

## Materials and methods

A retrospective chart review was conducted on all patients treated for glaucoma at the tertiary eye care health center, who had medically uncontrolled glaucoma following treatment using the Iridex MP-TSCPC and subsequently underwent SC-CWCPC between January 2022 and October 2024. This study was approved by the University at Buffalo Institutional Review Board and adhered to the tenets of the Declaration of Helsinki. Patients with baseline visual acuity better than no light perception, and a minimum of 3 months of follow-up were included. We chose the minimum of 3 months follow-up as prior studies reported failures occurring at this time point [9, 14]. 

Patients with a history of prior minimally invasive glaucoma surgery (MIGS), trabeculectomy, and tube shunts were included in this study. Patients with incomplete documentation of preoperative values such as IOP, anti-glaucoma medication (AGM), or visual acuity (VA) converted from Snellen to Logarithm of the Minimum Angle of Resolution (logMAR), history of ocular trauma, and congenital anterior chamber abnormalities were excluded from the study. Data was collected from Nextech Intellechart v8 Electronic Health Record regarding the patient demographics, type of glaucoma (primary open angle, neovascular, uveitic, congenital, juvenile glaucoma), preoperative VA, IOP and AGM’s (oral or topical), lens status, and prior history of glaucoma procedures (tubes, trabeculectomy, MIGS). The postoperative IOP and AGM were noted at postoperative day (POD)1, postoperative month (POM)1, POM3, POM6, POM12, and the last follow-up period. The success was defined as an IOP between 6 and 21 mmHg, and IOP reduced > 20% below baseline on their last follow up visit. Criteria for failure included if the patient had any subsequent incisional operation for glaucoma due to procedure failure or loss of light perception vision. We did not count additional treatment for SC-CWCPC as failure, which is consistent with prior studies [15]. 

The complications from the procedure including vision loss to no light perception, conjunctival burns, corneal decompensation, prolonged AC inflammation, hypotony, and cystoid macular edema were noted.

The consent was obtained for all patients undergoing SC-CWCPC procedure. Anesthesia was provided with monitored anesthesia care and topical lidocaine gel similar to prior studies [8, 9]. The initial MicroPulse laser was delivered with the CycloG6 transscleral diode laser using the Iridex revised P3 probe by a single glaucoma fellowship-trained surgeon (SS) with experience using MP-TSCPC (MicroPulse P3, Iridex Corporation, Mountain View, CA).

All patients subsequently underwent SC-CWCPC by the same surgeon (SS). A laser power of 1250 mW and 4 s duration per application was used, similar to other studies [11]. The average number of spots applied per procedure was 11.40 ± 1.65 (range: 8–14). The diode laser was applied using the G-probe (Iridex Corporation, Mountain View, CA). The surgical technique used was similar to that as described by Gaasterland with one key variation, we did not adjust the laser power based on iris pigmentation. In contrast, Gaasterland used a laser power of 1250 mW for darkly pigmented eyes And 1500mW for lightly pigmented eyes [16]. 

Statistical Analysis was performed using GraphPad Prism version 10.4.2.633. The normality of the data was evaluated by Kolmogorov- Smirnov test. Descriptive statistics were used to describe the frequencies and distribution of demographic data. Parametric tests were employed. The paired t test was used to compare the pre- and post- intervention IOP and AGM at different visits. P values < 0.05 were considered statistically significant.

## Results

A total of 10 eyes were included in this study. Baseline characteristics were obtained for all patients. (Table 1) Eight patients with a mean age of 43.75 ± 24.59 years (range:18–77 years) met the eligibility criteria. 90% of eyes (9/10) had a prior Tube/Trabeculectomy/MIGS surgery.

**Table 1:** Baseline characteristics of patients undergoing slow wave cyclophotocoagulation

Baseline characteristics	
**Age (years)**	43.75 ± 24.59
**Gender**,** N (%)**	Male: 4 (50) Female: 4 (50)
**Race**,** N (%)**	White: 4 (50) Declined to state: 4 (50)
**Eye**,** N (%)**	Right eye: 4 (40) Left eye: 6 (60)
**Lens Status**,** N (%)**	Phakic: 6 (60) Pseudophakic: 4 (40)
**Type of Glaucoma**,** N (%)**	Primary open Angle glaucoma: 4 (40) Juvenile glaucoma: 2 (20) Congenital glaucoma: 2 (20) Neovascular glaucoma: 1 (10) Uveitic glaucoma: 1 (10)
**Number of Laser Spots**	11.4 ± 1.65
**Baseline Visual Acuity (logMAR)**	1.58 ± 0.40
**Baseline Intraocular Pressure (mmHg)**	26.1 ± 6.3
**Baseline Anti-Glaucoma Medications**	3.1 ± 1.2
**Humphrey Visual Field** **Mean Deviation (dB)**	−19.86 ± 10.07
**Previous Surgeries**,** N (%)**	Prior Tube: 9 (90) Prior Trab: 3 (30) Prior MIGS: 1 (10)
**Follow-up (months)**	10.97 ± 3.4

**Fig. 1:**
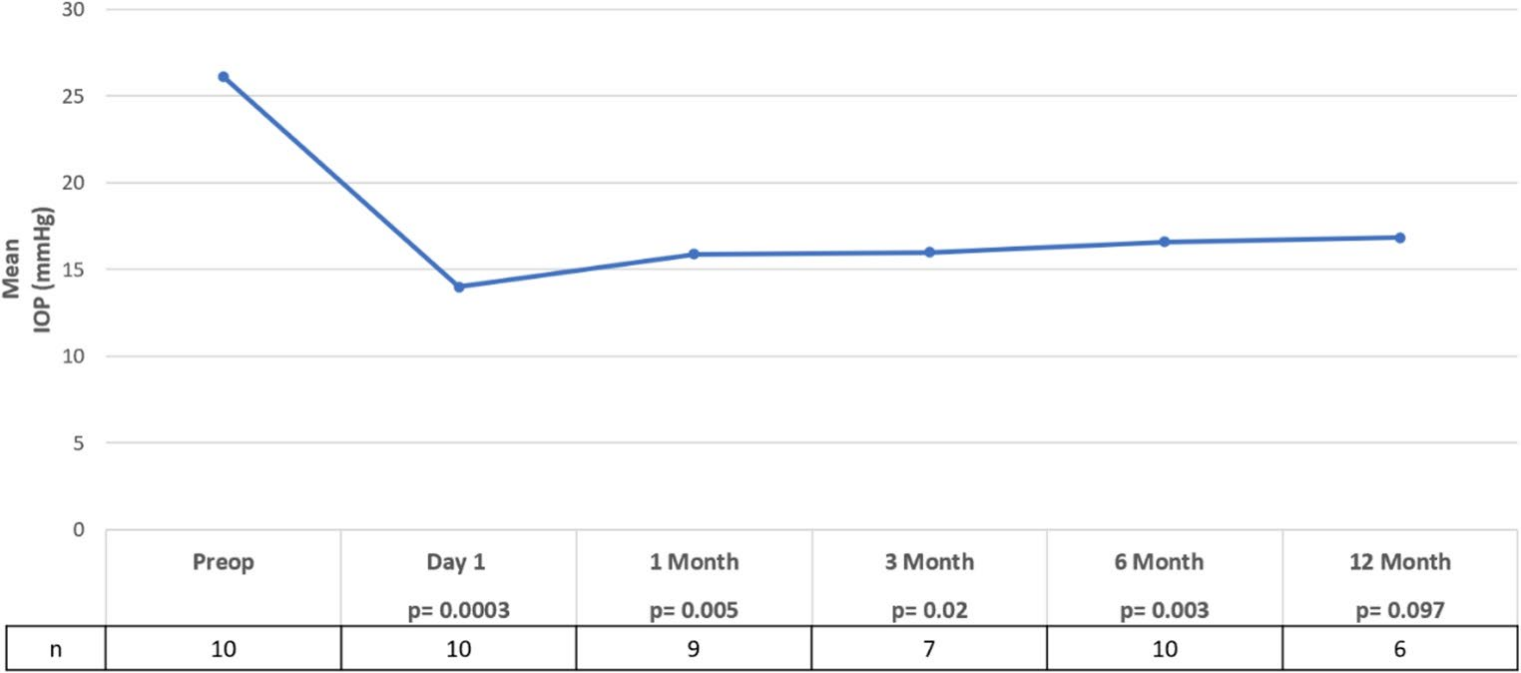
Average intraocular pressure (IOP) measured at baseline and post operatively following slow coagulation continuous wave cyclophotocoagulation. IOP showed a significant reduction compared to baseline at all time points excluding postoperative month 12 (*p* < 0.05)

There was a significant reduction in mean IOP from 26.10 ± 6.26 mmHg preoperatively to 15.80 ± 5.31 mmHg (*p* = 0.002) postoperatively at a mean follow-up of 10.97 ± 3.42 months. (Fig. 1) IOP was significantly reduced compared to baseline at all time points except POM12. One eye (uveitic glaucoma) required Ahmed Glaucoma Valve implantation at 11 months, while two eyes (congenital glaucoma and POAG) underwent repeat SC-CWCPC with one requiring a single retreatment at POM14 and the other requiring two retreatments at POM10 and POM11.

**Fig. 2:**
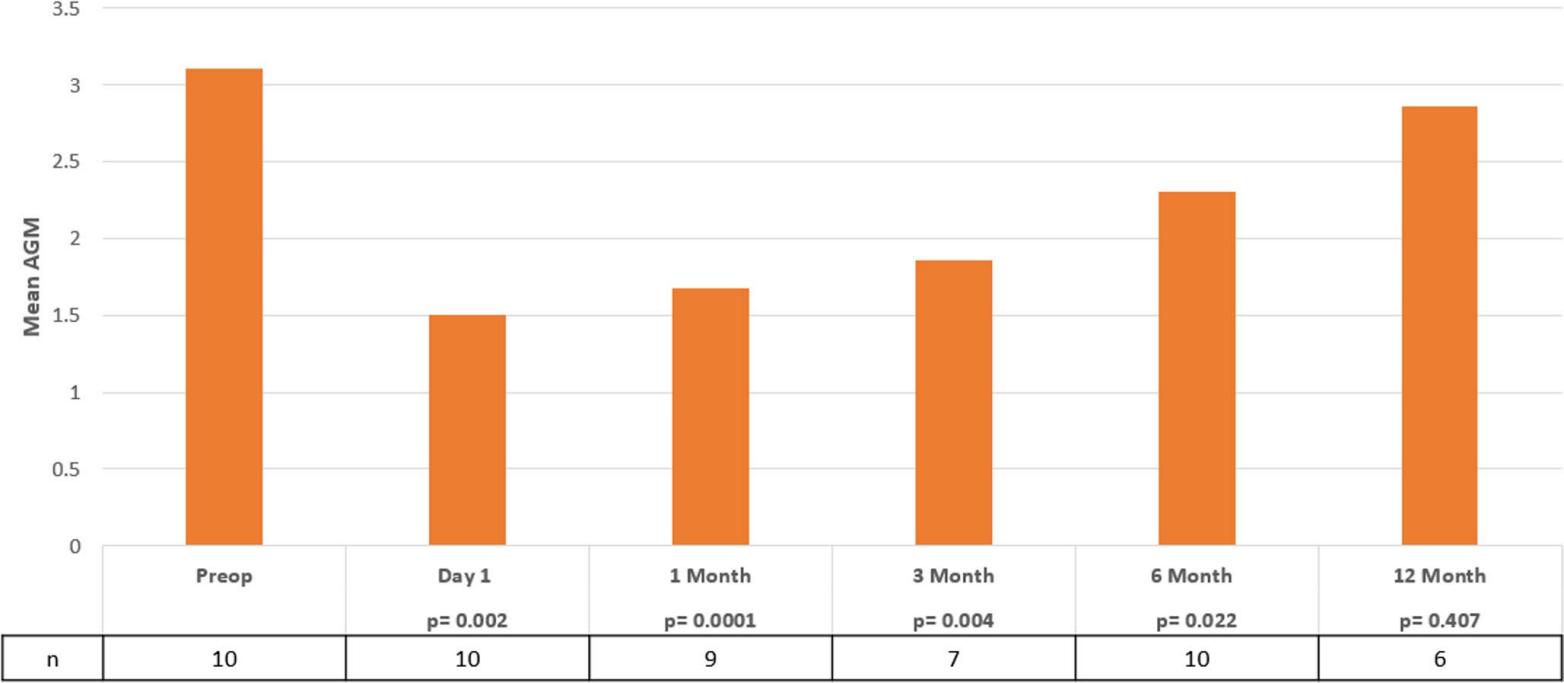
Average antiglaucoma medications (AGM) measured at baseline and post operatively following slow coagulation continuous wave cyclophotocoagulation. AGM showed a significant reduction compared to baseline at all time points excluding postoperative month 12 (*p* < 0.05)

The number of AGMs was also evaluated at pre- and post-operative visits. (Fig. 2) Patients treated with SC-CWCPC had a significant reduction in mean AGM compared to baseline at POD1, POM1, POM3, and POM6 however, this effect was not maintained past that time point. AGM decreased from 3.1 ± 1.20 to 2.30 ± 1.45 at the final follow up visit at 10.97 ± 3.42 months p=(0.070) with one eye not requiring AGM postoperatively. The mean logMAR changed from 1.58 ± 0.40 preoperatively to 1.60 ± 0.34 at the last follow up which was statistically insignificant p=(0.547). In 8 eyes, no change from baseline VA occurred, one eye improved by one line while another worsened by one line.

The overall success rate at POM3, POM6, and POM12 were 71%, 80%, and 67% respectively. Lastly, complications following SC-CWCPC were assessed which included one patient (1 eye) with prolonged AC inflammation managed with topical corticosteroids and resolved within 2 months, and another patient (both eyes) with corneal decompensation at POM1. It is worth noting however, that this patient had a significant history of corneal disease prior to the SC-CWCPC procedure (Right: prior penetrating keratoplasty (PKP), descemet stripping automated endothelial keratoplasty (DSAEK), and a repeat DSAEK; Left: DSAEK) with increased pachymetry of the right eye (CCT 966) and normal for the left (CCT 555). The right eye required a repeat PKP at POM11 while the left eye has remained on Muro 128. Given the patient’s history, it is most likely that his corneal decompensation was attributed to progression of his underlying corneal disease rather than from SC-CWCPC directly. Notably, his corneal edema may have led to an underestimation of IOP during follow-up however it remained stable nonetheless.

## Discussion

This case series found SC-CWCPC to be an effective and relatively safe surgical intervention in patients with uncontrolled refractory glaucoma. To our knowledge, this is the first study analyzing the efficacy of SC-CWCPC in recalcitrant glaucoma patients following a prior failed MP-TSCPC treatment.

Our study found an 80% (8/10) success rate at a mean follow-up visit of 10.97 months (median:11.3), suggesting that SC-CWCPC may be an effective surgical option for patients with uncontrolled refractory glaucoma following a failed MP-TSCPC. A study evaluating repeat MP-TSCPC, reported success rates of 36.4%, 42.9%, and 32.0% at postoperative years 1, 2, And 3, respectively [15]. Another study comparing SC-CWCPC vs. MP-TSCPC found success rates after a single session at 12 months to be 50.1% vs. 38.2%, however this difference was not significant [12]. Our study found a higher success rate of 67% (4/6 eyes) at postoperative year 1, however a key difference with the findings of this study compared to ours was that we did not consider retreatment as a failure, with two eyes undergoing repeat SC-CWCPC at POM 10 and 14. Our results highlight the efficacy of SC-CWCPC in patients with particularly challenging forms of glaucoma (Neovascular, congenital, and uveitic subtypes) further highlighting its versatility across a broad spectrum of complex disease presentations.

The higher success rate observed in our study may be due to the unique benefits SC-CWCPC theoretically offers, such as better-controlled tissue ablation and decreased collateral tissue damage and inflammation [17]. Studies evaluating the success rate of traditional CPC in the setting of refractory glaucoma have found success to range from 37% to 80% with differences in success criteria [18]. One study found a direct correlation between success rate (IOP < 22 mmHg) and total energy delivered [19]. Compared to traditional CWCPC which utilizes power ranging from 1500 to 2500mW, SC-CWCPC utilizes powers of 1250 to 1500mW and does not produce the characteristic pop that is heard which is thought to be due to micro-destructive explosive damage to the ciliary body [17]. However, if a pop is heard, this is likely due to the poor positioning of the probe, which should resolve when corrected [11]. These findings may presumptively lead one to think CWCPC remains the superior option. However, studies have demonstrated that the slow-coagulation technique and the characteristic “pop” technique are comparable with decreased complication risk in patients undergoing slow-coagulation technique [11, 17]. 

Our study found a significant reduction in AGM that was short lived and not maintained beyond 6 months. Prior studies have suggested that regeneration of the ciliary body can occur following treatment [20–22]. If more aggressive treatment protocols are capable of reducing ciliary body regeneration, then this could help explain the transient decrease in AGM followed by the reintroduction of AGM in our patients, however this hypothesis remains uncertain [9]. Importantly, we found no significant changes to VA among our patients. Eight patients (80%) did not experience any change from their baseline VA, while one patient (10%) improved a line of vision, and another (10%) experienced a one-line decrease. Our findings are similar to prior studies looking at SC-CWCPC who have found no significant changes to VA, with 13% of patients (7/53) demonstrating a decline compared to baseline [10]. All of the complications observed in our study were self-limiting with no long-term sequelae. One patient experienced prolonged AC inflammation while two patients demonstrated corneal decompensation.

When considering cost-effectiveness, the earlier use of SC-CWCPC following failed incisional surgery may be advantageous compared to the more expensive options such as MIGS or implantation of a second glaucoma drainage device [23, 24]. Additionally, SC-CWCPC is a relatively non-invasive procedure that does not limit future surgical interventions and can be performed in an office or operating room [25]. One study looking at SC-CWCPC in patients with no prior history of treatment found success ranging between 28.1 and 58.3% with a higher degree of success in those with a preoperative IOP > 21 mmHg [26]. All these features suggest that SC-CWCPC may serve as an effective early treatment option in the management glaucoma; however further studies are needed to establish its efficacy.

Some limitations of our study include the retrospective nature of our study which may introduce potential biases. Additionally, our small sample size along with a relatively short follow-up time may limit the generalizability of our findings. Further larger scale prospective studies are required to examine the efficacy of SC-CWCPC for treatment refractory glaucoma and the long-term efficacy.

## Conclusion

Our study found SC-CWCPC to be an effective and relatively safe surgical intervention in medically uncontrolled glaucoma refractory to prior MP-TSCPC and incisional surgeries. Patients who underwent SC-CWCPC demonstrated significant IOP reductions while maintaining VA and experiencing a low rate of complications. These findings support the expanded use of the slow-coagulation CW-TSCPC approach in the management of complex glaucomatous cases.

## Data Availability

Data is provided within the manuscript.
